# Comparison of the virulence of exopolysaccharide-producing *Prevotella intermedia *to exopolysaccharide non-producing periodontopathic organisms

**DOI:** 10.1186/1471-2334-11-228

**Published:** 2011-08-25

**Authors:** Takeshi Yamanaka, Kazuyoshi Yamane, Tomoyo Furukawa, Chiho Matsumoto-Mashimo, Chieko Sugimori, Takayuki Nambu, Noboru Obata, Clay B Walker, Kai-Poon Leung, Hisanori Fukushima

**Affiliations:** 1 Department of Bacteriology, Osaka Dental University, 8-1 Kuzuha-Hanazono, Hirakata, 573-1121 Japan; 2 Department of Oral Biology, College of Dentistry, University of Florida, Box 100424 UF Health Science Center, Gainesville, FL 32610-0424, USA; 3 US Army Dental and Trauma Research Detachment, Institute of Surgical Research, 3650 Chambers Pass, Fort Sam Houston, TX 78234-6315, USA

## Abstract

**Background:**

Evidence in the literature suggests that exopolysaccharides (EPS) produced by bacterial cells are essential for the expression of virulence in these organisms. Secreted EPSs form the framework in which microbial biofilms are built.

**Methods:**

This study evaluates the role of EPS in *Prevotella intermedia *for the expression of virulence. This evaluation was accomplished by comparing EPS-producing *P. intermedia *strains 17 and OD1-16 with non-producing *P. intermedia *ATCC 25611 and *Porphyromonas gingivalis *strains ATCC 33277, 381 and W83 for their ability to induce abscess formation in mice and evade phagocytosis.

**Results:**

EPS-producing *P. intermedia *strains 17 and OD1-16 induced highly noticeable abscess lesions in mice at 10^7 ^colony-forming units (CFU). In comparison, *P. intermedia *ATCC 25611 and *P. gingivalis *ATCC 33277, 381 and W83, which all lacked the ability to produce viscous materials, required 100-fold more bacteria (10^9 ^CFU) in order to induce detectable abscess lesions in mice. Regarding antiphagocytic activity, *P. intermedia *strains 17 and OD1-16 were rarely internalized by human polymorphonuclear leukocytes, but other strains were readily engulfed and detected in the phagosomes of these phagocytes.

**Conclusions:**

These results demonstrate that the production of EPS by *P. intermedia *strains 17 and OD1-16 could contribute to the pathogenicity of this organism by conferring their ability to evade the host's innate defence response.

## Background

Exopolysaccharide (EPS) productivities in many bacteria have been associated with pathogenicity in mammalian hosts as providing extracellular matrices to form biofilm or capsular polysaccharides attached to the cell surface [1-3]. Within biofilms, bacteria themselves are embedded in EPS and organise as a multicellular community [4]. Many gram-positive and gram-negative bacteria also produce polysaccharides that remain attached to the cell to form a capsule [5,6]. Some clinical isolates of *Prevotella intermedia *and *Prevotella nigrescens *produce mannose-rich EPSs. As revealed by electron microscopy, these clinical isolates showed dense meshwork structures around their cells [7,8], which are similar to the phenotype of other biofilm-forming bacteria such as *Pseudomonas aeruginosa *[9], salmonellae [10,11], *Escherichia coli *[12,13], and *Vibrio cholerae *[14]. Like the mucoid type of *P. aeruginosa *[15], these clinical isolates of *P. intermedia *spontaneously produce EPS and form meshwork structures around their cells, even under planktonic growth conditions [8]. EPS productivity is known to enhance the virulence properties of otherwise innocuous or relatively low virulent bacteria [7,8,16,17]. To determine the role of EPS for the expression of virulence in *P. intermedia*, we compared clinical isolates of EPS-producing *P. intermedia *to the reference strain *P. intermedia *ATCC25611 and EPS non-producing strains of *Porphyromonas gingivalis*, the organism that is most strongly associated with periodontal diseases.

## Methods

### Bacterial strains and cultures

EPS-producing *P. intermedia *17 (*Pi*17) [8,18] and OD1-16 (*Pi*OD1-16: a viscous material-producing clinical isolate from a chronic periodontitis lesion identified by 16S rRNA gene sequencing) were used in this study. We examined the chemical composition of viscous materials in *Pi*OD1-16 culture supernatant and confirmed that this organism produced mannose-rich EPSs whose chemical composition is similar to those of *Pi*17 [8] (see below). *P. intermedia *ATCC 25611 (*Pi*25611), *P. gingivalis *ATCC 33277 (*Pg*33277), W83 (*Pg*W83) and 381 (*Pg*381) were used as reference strains to compare the bacteria's capacity to induce abscess formation in mice. These bacterial strains were grown on trypticase soy blood agar plates supplemented with 0.5% yeast extract (Difco Laboratories, Detroit, MI), hemin (5 mg/l), L-cystine (400 mg/l), and vitamin K_1 _(10 mg/l) (enriched BAP) or grown in trypticase soy broth (BBL Microbiology Systems, Cockeysville, ND) supplemented with 0.5% yeast extract, hemin (5 mg/l), L-cystine (400 mg/l), and vitamin K_1 _(10 mg/l) (enriched TSB). Bacterial cultures were grown anaerobically in an anaerobic chamber (ANX-3, Hirasawa, Tokyo, Japan) at 37°C in a 5% CO_2_, 10% H_2_, 85% N_2 _atmosphere. Growth of the tested organisms in enriched TSB was monitored by following the optical density at 600 nm with U-2000 spectrophotometer (Hitachi, Tokyo, Japan).

### Viscosity of spent culture media, cell surface structures and chemical compositions of viscous materials

The ability to produce EPS in liquid culture media and form meshwork structures on cell surfaces by the test bacteria was examined as described elsewhere [8]. Briefly, the stock strains were grown in enriched TSB for 48 h. The spent culture medium (550 μl) was put into a rotor, and the viscosity was measured as shearing stress between a rotor and a rotor shaft at 50 rpm, 20°C using a rotary viscometer (Toki-sangyo, Tokyo, Japan). Five independent cultures of each strain were measured and statistical differences between the bacterial cultures and the control medium were determined using an unpaired *t*-test with the level of significance set at P < 0.05.

To examine cell surface structures, scanning electron microscopy (SEM) was performed. Bacteria grown on enriched BAP for 48 h were collected on a piece of filter paper (Glass fiber GA55, Toyo Roshi, Tochigi, Japan), fixed with 2% glutaraldehyde in 0.1 M phosphate buffer (PB) for 2 h and 1% OsO_4 _in 0.1 M PB for 1 h at 4°C, and dehydrated through an ethanol series and 2-methyl-2-propanol followed by platinum ion coating (E-1030, Hitachi, Tokyo, Japan). Specimens were examined with a scanning electron microscope (S-4800, Hitachi) at an accelerating voltage of 3 kV.

The EPS was prepared from culture supernatants as described [8]. In brief, OD1-16 was grown at 37°C in enriched TSB for 48 h. Supernatants were separated by centrifuging the liquid culture at 12,000 × *g *for 30 min, and sodium acetate was added to a final concentration of 5%. The mixture was stirred for 30 min at 22°C, and the EPS was isolated by ethanol precipitation from the reaction mixture. The ethanol-precipitated material was collected by centrifugation (18,200 × *g *for 15 min at 22°C), resolved in 5% sodium acetate, and treated with chloroform: 1-butanol (1: 5 by volume). Water-soluble and chloroform-butanol layer were separated by centrifugation, an equal amount of ethanol was added to the water-soluble layer (this procedure was repeated twice), and the ethanol-precipitated material was freeze-dried and stored at -80°C until use [19]. Contaminated lipopolysaccharides were removed from preparations by the method described by Adam *et al*. [20].

The sugar composition of the purified viscous material were determined by means of high performance liquid chromatography (HPLC) for neutral and amino sugars and colorimetry for uronic acid as detailed elsewhere [8].

### Animal studies

The virulence of EPS-producing clinical isolates of *P. intermedia *was compared with those of *Pi*25611, *Pg*33277, *Pg *W83 and *Pg *381. Bacterial strains were cultured in enriched TSB for 32 h for *Pi*17 and *Pi*OD1-16 and for 24 h for *Pi*25611, *Pg*33277, *Pg*W83 and *Pg*381 (early stationary phase). The inguen of each BALB/c mouse (male, 4 weeks; 5 mice per strain) was injected subcutaneously with 500 μl of bacterial suspensions (10^7 ^to 10^10 ^CFU). Changes of abscess lesions were recorded photographically with a camera set (Nikon FIII, Nikon, Japan) at a fixed magnification for 5 consecutive days. Abscess areas recorded on the third day were measured and analyzed with image analysis software (Image Measure; Imsoft, Tokyo, Japan). The experiments were performed twice. The animal studies were done according to the guidelines for animal experimentation at Osaka Dental University.

### Phagocytosis assay

To compare antiphagocytic activity among the strains used in this study, bacterial cells were co-cultured with polymorphonuclear leukocytes (PMNLs) obtained from healthy human volunteers (n = 3; age 20-23 years) as described previously [8]. After incubation for 90 min, PMNLs co-cultured with bacterial cells were centrifuged; and cell pellets were fixed with 2% glutaraldehyde in 0.1 M PB for 2 h at 4°C, post-fixed with 1% OsO_4 _in 0.1 M PB for 1 h at 4°C, and dehydrated through an ethanol series. Samples were embedded in epoxy resin and cut at 60-80 nm using an Ultracut (Leica, Tokyo, Japan). The sections were placed on a copper grid, contrasted with uranyl acetate and lead citrate, and examined under an H-7100 transmission electron microscope (Hitachi, Tokyo, Japan).

### Localization of bacteria *in vivo*

To examine the localization of bacterial cells *in vivo*, 10^8 ^CFU of *Pi*OD1-16, *Pi*25611 and *Pg*33277 were injected into the inguinal region of mice as described above. Mice were sacrificed at 48 h after the injection, and the regions where bacterial cell suspension was given were dissected under a stereoscopic microscope (Nikon, Tokyo, Japan). The specimens were cut into rectangular strips measuring 2 × 2 × 5 mm and fixed by immersion fixation in 2% glutaraldehyde for 2 h at 4°C, rinsed three times with 0.1 M PB, and post-fixed with 1% OsO_4 _in 0.1 M PB. Ultrathin sections were prepared for transmission electron microscopy **(**TEM) as described above.

## Results

### Viscosity of spent culture media and cell surface meshwork structures

**Figure 1:**
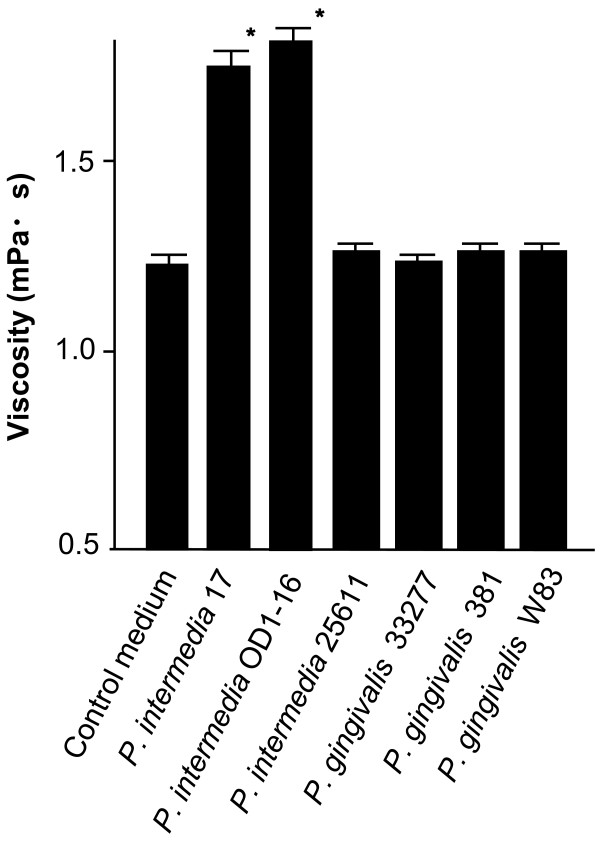
**Viscosity of the spent culture media obtained from *Prevotella intermedia *strains 17, OD1-16 and ATCC 25611 and *Porphyromonas gingivalis *strains ATCC 33277, 381 and W83**. Viscosity of the enriched trypticase soy broth was measured as a control. Bars indicate standard deviations. *P < 0.05.

**Figure 2:**
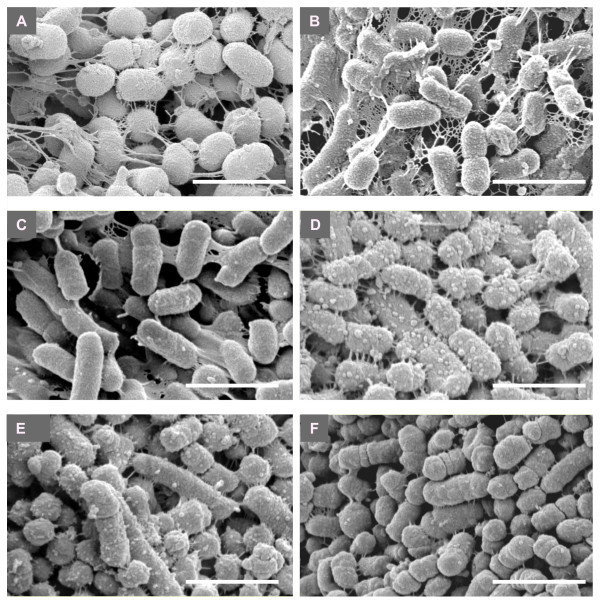
**Scanning electron micrographs showing surface structures of *Prevotella intermedia *strains 17 (A), OD1-16 (B) and ATCC 25611 (C) and *Porphyromonas gingivalis *ATCC 33277 (D), 381 (E) and W83 (F) grown on blood agar plates**. *P. intermedia *strains 17 and OD1-16 had dense meshwork structures surrounding the cell surfaces (A and B), but other strains lacked this phenotype. Bars = 1.2 μm.

**Figure 3:**
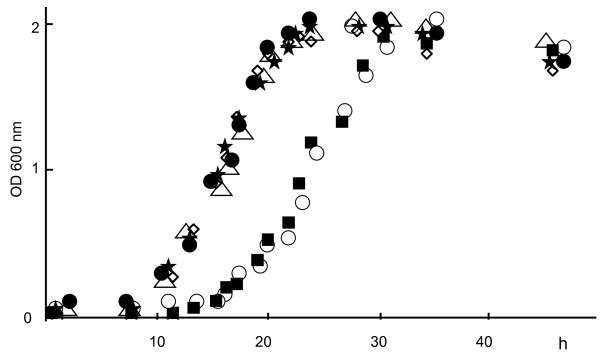
**Growths of test strains**. *Prevotella intermedia *strains 17 (black square) and OD1-16 (open circle) showed a slower growth rate, entering into an exponential phase at around 18 h and reaching the plateau in 32 h, than those of *P. intermedia *ATCC 25611 (open rhombus), *Porphyromonas gingivalis *ATCC 33277 (black star), 381 (open triangle) and W83 (black circle).

Our stock strains of EPS-producing *Pi*17 and *Pi*OD1-16 showed higher viscosity in their spent culture media than those of other periodontopathic bacteria used in this study (Figure 1). The viscosity of the spent culture media of *Pi*25611, *Pg*33277, *Pg*381 and *Pg*W83 was similar to that of the control medium without bacterial inoculation (Figure 1). SEM observation revealed that *Pi*17 and *Pi*OD1-16 maintained the ability to form dense meshwork structures around their cells (Figure 2). Typical meshwork structures were not observed around other strains although *Pi*25611, *Pg*33277 and *Pg*381 showed intercellular net structures (Figure 2). Growth of strains with EPS production (*Pi*17 and *Pi*OD1-16) was slower than that of EPS non-producing strains. *Pi*17 and *Pi*OD1-16 entered into exponential phase at around 18 h and reached plateau (stationary phase) in 32 h. Other strains that did not produce viscous material showed faster growth, entering into an exponential phase at 12 h and reaching plateau in 24 h (Figure 3).

### Chemical compositions of *Pi*OD1-16 EPS

Chemical analyses of the purified EPS from *Pi*OD1-16 cultures showed that it primarily consisted of neutral sugars and small amounts of uronic acid and amino sugars, with mannose constituting 82% of the polysaccharides (Table 1).

**Table 1:** Amount of neutral sugar, neutral sugar components, uronic acid and amino-sugar in the viscous material isolated from culture supernatants of *Prevotella intermedia *OD1-16

Sugar	Amount (μg/mg)
Neutral sugar	791.7
Mannose	682.1
Glucose	50.1
Galactose	30.3
Arabinose	16.4
Xylose	7.9
Uronic acid	27.7
Aminosugar	13.7

### Abscess induction in mice

**Figure 4:**
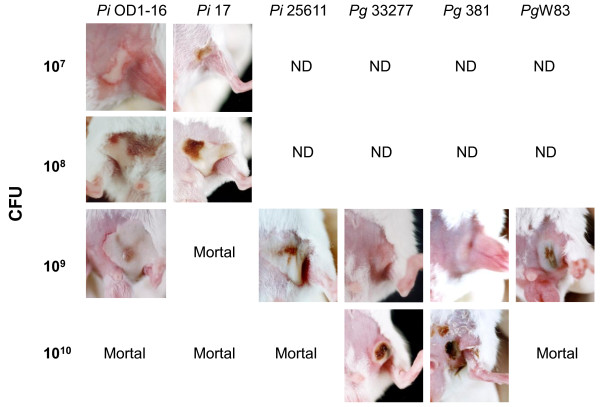
**Abscess induction in mice**. Abscess formation was induced when 0.5 ml of bacterial cell suspension (2 × 10^7 ^CFU/ml) of *Prevotella intermedia *strains 17 and OD1-16 was injected into the inguinal area of a mouse. In contrast, subcutaneously injected *P. intermedia *ATCC 25611, *Porphyromonas gingivalis *ATCC 33277, 381 and W83 (0.5 ml at a concentration of 2 × 10^8 ^CFU/ml) failed to induce an abscess in the mice. When these strains were injected at a higher concentration (2 × 10^9 ^CFU/ml), abscess formation was ultimately induced. The data are representative from one of two independent experiments.

The abilities of *Pi*17 and *Pi*OD1-16 to induce abscesses in mice were compared to those of other laboratory reference strains. The injections of 10^7 ^CFU of *Pi*17 and *Pi*OD1-16 induced abscesses in mice, respectively (Figure 4). In contrast, injections of a similar amount of *Pi*25611, *Pg*33277, *Pg*381 and *Pg*W83 at the same growth phase failed to induce abscesses in mice. A much higher cell concentration (10^9^-10^10 ^CFU) was required to induce abscesses in mice for these reference strains (Figure 4). The bacteria dose of 10^10 ^CFU was lethal at 24 h after the injection for all strains except *Pg*33277 and *Pg*381 (Table 2).

**Table 2:** Abscess area and lethality

Bacteria	Mean abscess area ± standard deviation^1 ^and lethality^2^			
	
	Infectious dose (CFU)			
	**10^7^**	**10^8^**	**10^9^**	**10^10^**

*Prevotella intermedia*				
Strain 17	19.5 ± 10.9 (0/10)	102.9 ± 56.1 (0/10)	Lethal (10/10)	Lethal (10/10)
OD1-16	15.9 ± 5.9 (0/10)	87.0 ± 59.5 (0/10)	292.3 ± 79.7 (8/10)	Lethal (10/10)
ATCC 25611	ND (0/10)	ND (0/10)	222.9 ± 83.5 (2/10)	Lethal (10/10)
*Porphyromonas gingivalis*				
ATCC 33277	ND (0/10)	ND (0/10)	191.7 ± 54.7 (0/10)	237.9 ± 59.8 (4/10)
381	ND (0/10)	ND (0/10)	242.1 ± 72.5 (0/10)	278.6 ± 122.5 (4/10)
W83	ND (0/10)	ND (0/10)	259.5 ± 97.4 (4/10)	Lethal (10/10)

^1^Abscess area was measured at 3 days after the injection.

^2^Motality of mice 24 h after the injection is shown in parentheses.

### Phagocytosis assay with human PMNLs

**Figure 5:**
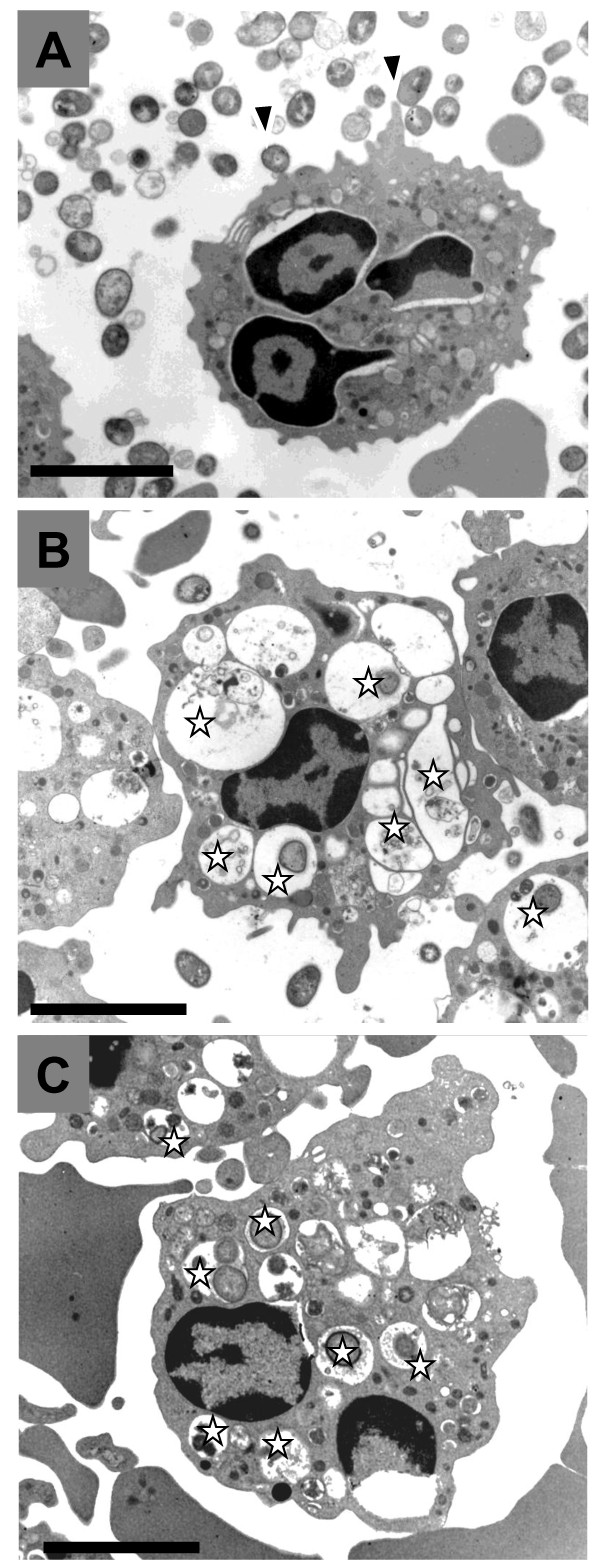
**Resistance of viscous material-producing *Prevotella intermedia *strain OD1-16 against the phagocytic activity of human polymorphonuclear leukocytes *in vitro***. *P. intermedia *OD1-16 cells were observed around the periphery of polymorphonuclear leukocyte but not internalized (A, arrow heads). In contrast, *P. intermedia *ATCC 25611 and *Porphyromonas gingivalis *ATCC 33277 cells without viscous material production were readily internalized and the ingested bacteria appear to be enclosed in cytoplasmic vacuoles (B and C, open stars). The data are representative from one of three independent experiments. Bars = 3.3 μm.

**Figure 6:**
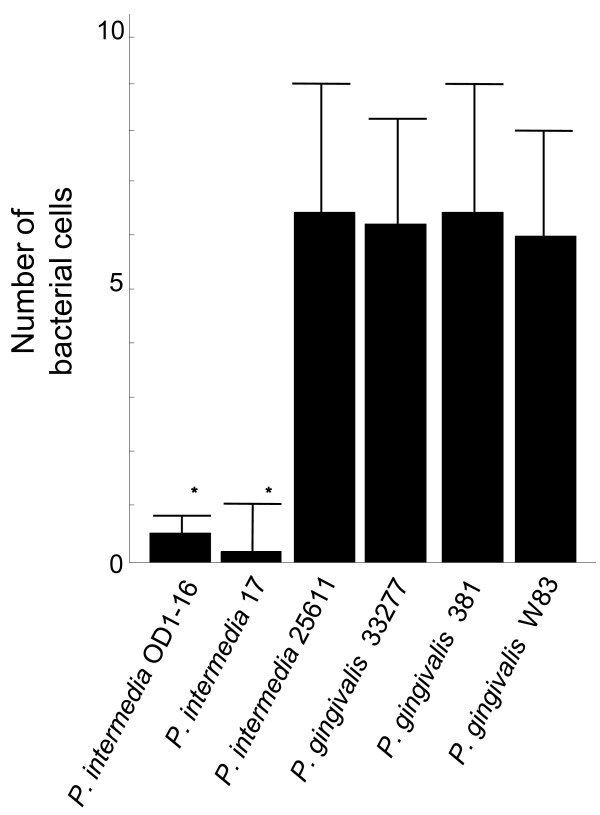
**Number of bacterial cells engulfed in a polymorphonuclear leukocyte (PMNL)**. Under transmission electron microscopy (TEM), 30 PMNLs were arbitrarily selected, and the number of bacterial cells engulfed in each cell was counted. The results show the average number and standard deviation for the number of bacterial cells engulfed in each PMNL. Statistical difference to *Prevotella intermedia *ATCC 25611 was determined using unpaired *t*-test with the significance set at P < 0.05 (*).

Next, we addressed whether *P. intermedia *with EPS production is resistant to phagocytosis by human PMNLs *in vitro*. In contrast to *Pi*17 and *Pi*OD1-16 that were rarely internalized, other test strains were readily internalized by human PMNLs, namely six bacterial cells on an average were found in cytoplasmic vacuoles of each PMNL as revealed by TEM (Figure 5 and 6).

### Localization of bacteria *in vivo*

**Figure 7:**
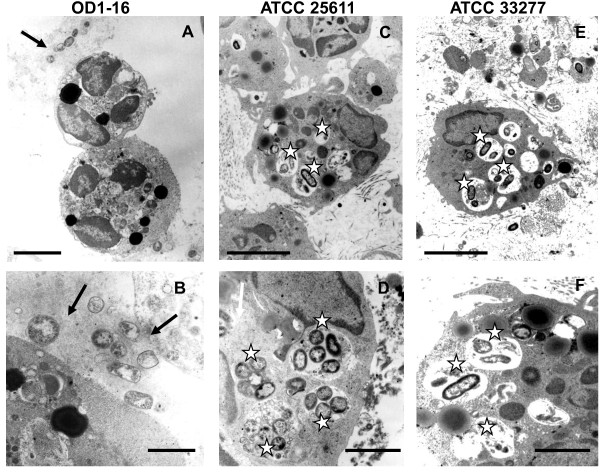
**Resistance of viscous material-producing *Prevotella intermedia *strain OD1-16 against the phagocytic activity of murine polymorphonuclear leukocytes (PMNLs) *in vivo***. *P. intermedia *OD1-16 cells were not internalized by PMNLs (A and B, arrows). In contrast, *P. intermedia *ATCC 25611 and *Porphyromonas gingivalis *ATCC 33277 cells were ingested and digested in phagolysosomes (C-F, open stars). The data are representative from one of two independent experiments. Bars = 3.3 μm (A, C, and E) and 1.2 μm (B, D, and F).

To further investigate the antiphagocytic effect of EPS *in vivo*, 10^8 ^CFU of *Pi*OD1-16, *Pi*25611 and *Pg*33277 were injected into inguinal regions of mice, and the localization of bacterial cells was observed by TEM. In the murine tissues, bacterial cells of *Pi*OD1-16 were observed around PMNLs but rarely internalized (Figure 7A and 7B). In contrast, *Pi*25611 and *Pg*33277 cells were readily internalized and localized inside phagosomes (Figure 7C to 7F).

## Discussion

*P. intermedia *and the related *P. nigrescens *are known as periodontopathic bacteria and frequently isolated not only from various types of periodontal diseases [21-23] but infections in infants [24] and sinusitis lesions of dental origin [25]. Previously, we reported that clinical strains of *P. nigrescens *[7] and *P. intermedia *[8] isolated from chronic periodontitis lesions produce viscous materials under planktonic growth conditions. These materials form dense meshwork structures on cell surfaces as illustrated by SEM. Chemical analyses of the viscous materials isolated from their culture supernatants revealed that they consisted of mostly neutral sugars with mannose constituting more than 80% of the polysaccharide. Purified EPS itself did not exhibit any pathogenicity or immunogenicity. However, whole cells of clinical strains capable of producing EPS, in comparison to EPS non-producing mutants, show a strong ability to induce abscess in mice [7,8]. Interestingly, the virulence of the mutants to induce abscess in mice can be restored by co-application of the variants with the purified EPS [7]. Therefore, EPS could represent a key component contributing to the virulence of *P. intermedia *and *P. nigrescens*. Our finding is consistent with the findings of *P. aeruginosa *as reported previously [26]. In this regard, Deighton *et al*. [17] compared the virulence of slime-positive *Staphylococcus epidermidis *with that of a slime-negative strain in a mouse model of subcutaneous infection and showed that biofilm-positive strains produce significantly more abscesses and persisted longer in the infection.

To further evaluate the level of pathogenicity on the clinical strains of *P. intermedia *with EPS productivity, this study compared the ability of EPS-producing *P. intermedia *and several laboratory reference strains of periodontopathic bacteria (*Pi*25611, *Pg*33277, *Pg*381 and *Pg*W83) to induce abscess formation in mice. EPS-producing *Pi*17 and *Pi*OD1-16 induced abscess lesions in mice at 10^7 ^CFU, but other test periodontopathic bacteria did not when tested at this cell concentration. *Pi*25611 and *P. gingivalis *strains used in this study induced detectable abscess formation in mice when the infectious dose was 10^9 ^CFU and higher. The abscess model in mice with inoculum sizes of 10^9^-10^10 ^CFU has been used to demonstrate the biological activities of *P. gingivalis *[27,28]. Accordingly, the pathogenicity of *Pi*17 and *Pi*OD1-16 appeared to be stronger than those of the *P. gingivalis *strains as well as the ATCC strain of *P. intermedia *used in this study.

A wide range of microorganisms is known to produce EPS as a main constituent of the biofilm extracellular matrix, and recent investigations have revealed that each biofilm-forming bacterium produces distinctive EPS components [29]. In oral microbiota, *Capnocytophaga ochracea *found in the human oral cavity has been shown to produce mannose-rich EPS that can suppress murine lymphocyte mitogen responses and activate human complement response [30-32]. Kapran *et al*. [33] reported that *Aggregatibacter actinomycetemcomitans *has a gene cluster which is homologous to *E. coli pgaABCD *and encodes the production of poly-ß-1,6-GlcNAc (PGA) [34]. *Rothia mucilaginosa *DY-18 [35] and *Escherichia hermannii *YS-11 [36] isolated from persistent apical periodontitis lesions produced EPS and exhibited cell surface meshwork structures. The meshwork structures of *E. hermannii *YS-11 disappeared when *wzt*, one of the ABC-transporter genes, was disrupted by transposon random insertion mutagenesis. Complementation of this gene to the transposant restored and dramatically augmented the formation of meshwork structures. Our studies using an abscess model in mice indicated that this EPS phenotype might be involved in the pathogenicity of this organism [36]. Likewise, as described above, EPS productivity could be associated with *P. intermedia *and *P. nigrescens *pathogenicity [7,8].

In our experience, more than 20% of clinically isolated *P. intermedia *strains showed viscous material productivity under planktonic growth conditions [18]. This ability was lost in the course of sequential *in vitro *passage. As a result, less than 2% of clinical isolates remained as viscous material-producing strains (data not shown). Therefore, it is important to note that laboratory reference strains do not always represent the original virulence properties as Fux *et al*. [37] previously pointed out. Early studies have pointed out the relation between bacterial pathogenicity and polysaccharide productivity on the reference strains used in this study. Okuda *et al*. [38] reported that *Pi*25611, *Pg*381 and *Pg*33277 had capsular structures around the cells and that the capsular polysaccharides extracted from *Pg*381 contained galactose and glucose as their major constituents. *Pg*W83 is known to produce capsular polysaccharides, and the genetic locus for capsule biosynthesis has been identified [39,40]. As discussed in these earlier studies [38,41-44], cell surface-associated polysaccharides could represent another set of virulence factors in addition to production of various proteolytic enzymes, contributing to the pathogenicity of *P. gingivalis *strains. In this study, we did not detect the presence of capsular polysaccharide or production of EPS in *P. gingivalis *strains. One possibility is that the tested *P. gingivalis *strains had lost their ability to produce capsular polysaccharides or EPS because of multiple *in vitro *passages of the organisms in the laboratory. Further, none of these strains regained capsular polysaccharides or EPS productivity through repetitive animal passages (data not shown). This could explain the less virulent characteristics displayed by the tested *P. gingivalis *strains shown in our animal virulence experiments.

As our [7,8] and other earlier studies [26,45] indicated, it is plausible that the antiphagocytic effect of EPS confers the ability to *P. intermedia *to induce abscess. It has been demonstrated that the slime or components of slime from *S. epidermidis *cultures could contribute to the delay of clearance of this organism from host tissues. Similarly, in the murine model of systemic infection, the deletion of *ica *locus necessary for the biosynthesis of surface polysaccharide of *Staphylococcus aureus *significantly reduces its virulence [45]. As described above, a study in the early 1970s clearly showed that the addition of slime from *P. aeruginosa *cultures to *E. coli *or *S. aureus *dramatically inhibited phagocytosis by leukocytes [26]. In this study, EPS-producing *Pi*17 and *Pi*OD1-16 cells were rarely internalized by leukocytes both *in vitro *and *in vivo*. Many of these bacteria were seen localized to the cell surface of PMNLs but failed to be ingested. In such a situation, PMNLs are known to produce a harmful effect to the surrounding tissue by elaborating a variety of degradative enzymes and oxygen radicals in an attempt to clear the invaded pathogens [46,47]. In contrast, the test laboratory reference strains of periodontopathic bacteria, lacking EPS production, were readily engulfed and digested in phagosomes of phagocytes.

Most pathogenic *P. gingivalis *strains exhibit a higher resistance to phagocytosis than less pathogenic strains do [48]. In our study, the *P. gingivalis *strains were readily phagocytosed. It has been documented that freshly isolated pathogenic strains of *P. gingivalis *could lose invasiveness as a result of repeated subcultures [44]. Therefore, it is reasonable to speculate that pathogenic *P. gingivalis *strains become less pathogenic and more susceptible to phagocytosis by PMNLs when they lose the ability to produce capsular or extracellular polysaccharides though we have to carefully investigate the possibility that multiple factors exist in the observed incapability to induce abscesses in mice. We have not been able to restore any of our working strains of *P. gingivalis' *ability to express cell surface-associated meshwork structures or the ability to spontaneously produce viscous material in spent culture media. It is still unclear whether *P. gingivalis *with capsule formation or EPS productivity exhibits similar or higher pathogenicity to those of *P. intermedia *strains with meshwork structures.

## Conclusions

The data obtained in this study suggest that the pathogenicity of *P. intermedia *with the ability to produce EPS might be stronger than those of the *P. gingivalis *strains as well as the ATCC strain of *P. intermedia *that lack the ability to produce viscous materials. It is important to point out that freshly isolated clinical strains are needed to re-evaluate the pathogenicity of periodontopathic bacteria isolated from the dental plaque or periodontal lesions because many virulence phenotypes expressed in natural environmental niches could be lost through multiple laboratory passages.

The authors declare that they have no competing interests.

## Authors' contributions

TY, TF and CM carried out the phenotype characterization and drafted the manuscript. TY, KY, CS, TN and NO performed animal studies and phagocytosis assays. CBW, KPL, and HF participated in the design of this study and drafted the manuscript. All authors read and approved the final manuscript.

## Pre-publication history

The pre-publication history for this paper can be accessed here:

http://www.biomedcentral.com/1471-2334/11/228/prepub
